# Characterization of Gastrointestinal Absorption of Salmon Milt‐Derived Oligodeoxyribonucleic Acids in Mice

**DOI:** 10.1155/jnme/2183675

**Published:** 2026-05-29

**Authors:** Fuyu Hayashi, Yusuke Masuo, Yuki Nishizawa, Ayaka Koike, Takahiro Ishimoto, Keisuke Kiriyama, Mica Fujita, Keita Sutoh, Yukio Kato

**Affiliations:** ^1^ Faculty of Pharmacy, Kanazawa University, Kanazawa, Japan, kanazawa-u.ac.jp; ^2^ Nutritional Development and Research, Fordays Co., Ltd., Nihonbashi, Japan; ^3^ Fordays Nutritional Research Center, Tokyo University of Agriculture and Technology, Koganei, Japan, tuat.ac.jp

**Keywords:** dideoxyribonucleotide, dietary DNA, intestinal absorption, LC–MS/MS, pyrimidine nucleoside, uric acid

## Abstract

Dietary nucleic acids exert physiological effects beyond serving as substrates for nucleic acid synthesis; however, their exact molecular forms that are absorbed in the small intestine remain unclear. In this study, we investigated the absorption forms of food‐derived DNA by comprehensively quantifying 31 nucleic acid monomers that permeated the intestinal membrane after oral administration, using LC–MS/MS. Hydrolyzed *Oncorhynchus* milt DNA (HD‐omDNA; mainly 1–4‐mer nucleotides), deoxyribonucleotides (dNTs), and deoxyribonucleosides (dNSs) were orally administered to mice, and blood samples were collected from the tail vein, aorta, and portal vein. Intestinal permeation was also assessed using the Ussing chamber method. Oral administration of HD‐omDNA, dNTs, or dNSs increased the plasma levels of pyrimidine dNSs—such as thymidine, deoxyuridine, and deoxycytidine—identifying them as the major forms of high‐molecular‐weight DNA absorbed into systemic circulation. Portal vein concentrations of these pyrimidine dNSs were elevated compared with vehicle controls, confirming their intestinal absorption. In contrast, purine nucleic acid monomers were metabolized to uric acid, as indicated by elevated portal vein concentrations of its precursors, hypoxanthine, and xanthine upon co‐administration with the xanthine oxidase inhibitor febuxostat. Notably, dinucleotides were also detected in portal vein plasma in vivo and on the basolateral side in Ussing chamber experiments, suggesting that dinucleotides are a previously unrecognized absorption form of HD‐omDNA. Collectively, these findings demonstrate that dietary DNA permeates the intestine mainly as pyrimidine dNSs, whereas dinucleotides are also an absorption form, revealing unique absorption characteristics of dietary deoxyribonucleotides and oligodeoxyribonucleotides.

## 1. Introduction

Nucleic acids are compounds that not only serve as the structural units of DNA and RNA but also play essential roles in bioenergetics and signal transduction. Nucleotides—the fundamental building blocks of polymeric nucleic acids—are synthesized via the de novo pathway. However, this process is energetically demanding—requiring six ATP molecules for each purine nucleotide and two ATP molecules for each pyrimidine nucleotide—making it inefficient to meet the body’s total requirement [1]. In addition to the de novo pathway, a salvage pathway exists that reutilizes the nucleic acid derivatives produced by the degradation of endogenous nucleic acids or obtained from dietary sources [2]. Dietary nucleic acids exert various physiological effects in addition to their incorporation into nucleic acids. For instance, *Oncorhynchus* milt DNA (omDNA), a nucleic acid fraction derived from salmon milt extract, is known to prevent alcoholic liver injury [3], while the intake of 2.5% omDNA has been reported to improve learning and memory in healthy mice [4]. Oral administration of omDNA has also been shown to reduce ascites volume in mice bearing Ehrlich ascites tumors [5]. These findings suggest that dietary nucleic acids may be absorbed through the gastrointestinal tract and distributed to target organs, such as the liver, brain, and even the pathological sites, where they exert functional effects. However, the exact molecular forms in which nucleic acids are absorbed and distributed in vivo remain unclear.

In humans, daily nucleic acid intake is estimated to be 0.1–1 g [6]. DNA is present in various food sources, with particularly high concentrations in meat products (5–100 g/kg dry matter) [6]. These nucleic acids are degraded in the gastrointestinal tracts into a mixture of mono‐, di‐, tri‐, and polynucleotides [1, 2]. Because of their high water solubility, nucleic acids are unlikely to permeate cellular membranes via passive diffusion, suggesting that transporter‐mediated uptake is essential for absorption across the intestinal epithelium. Nucleoside transporters, including concentrative nucleoside transporters (CNTs; SLC28 family) and equilibrative nucleoside transporters (ENTs; SLC29 family), facilitate the cellular uptake of nucleosides [7–10]. In addition, nucleobase‐specific transporters such as sodium‐dependent nucleobase transporter 1 (SNBT1/SLC23) and equilibrative nucleobase transporter 1 (ENBT1/SLC43A3) have been identified [11, 12]. Because some ENTs and CNTs are expressed in the intestinal tissues [13], it is generally assumed that nucleosides generated by hydrolysis are the primary absorbable forms. However, this assumption is based mainly on in vitro data and transporter expression profiles; the actual molecular species absorbed in vivo are yet to be elucidated.

To investigate gastrointestinal absorption and subsequent disposition of exogenous nucleic acids, previous studies have employed radiolabeled nucleic acids to distinguish them from their endogenous counterparts. In one study, mice were orally administered *E. coli* DNA labeled with radioactive thymine or yeast RNA labeled with radioactive adenine and guanine. Their results revealed that 6 h postadministration, ∼70% of the DNA‐derived radioactivity was recovered as CO_2_ in exhaled air and 15% in tissues, whereas ∼80% of the RNA‐derived radioactivity was detected in urine and 20% in tissues [14]. A major limitation of these approaches is that radioactivity measurements alone cannot differentiate between specific molecular forms. Given the complexity of nucleic acid metabolism and the diversity of its intermediates, previous studies did not provide a comprehensive understanding of their in vivo fates.

From a nutritional perspective, although it is plausible that nucleic acids are completely hydrolyzed into nucleosides and nucleobases before absorption, the possibility remains that partially degraded oligonucleotides or even high‐molecular‐weight nucleic acids may also be absorbed through the small intestine. Elucidating the molecular forms of absorbed nucleic acids would not only advance our understanding of their nutritional functions but also support the development of orally administered nucleic acid therapeutics. In the present study, we aimed to clarify the molecular forms of food‐derived DNA absorbed through the gastrointestinal tract by comprehensively quantifying nucleic acid‐derived compounds that permeate the intestinal membrane following oral administration.

## 2. Methods

### 2.1. Materials

Sodium salts of omDNA were extracted from salmon milt, precipitated with hydrochloric acid, and subsequently neutralized. The hydrolyzed omDNA (HD‐omDNA), which is possessed on the low molecular weight side, was then obtained by hydrolysis of omDNA with nuclease “Amano”G (Amano Enzyme Inc., Aichi, Japan) and other enzymes. Both omDNA and HD‐omDNA were obtained from Fordays Co. Ltd. (Tokyo, Japan). 2′‐deoxyadenosine‐5′‐monophosphate (dAMP) and 2′‐deoxythymidine‐5′‐monophosphate (dTMP) were purchased from Chem‐Impex International (IL, UA). 2′‐deoxyguanosine 5′‐monophosphate (dGMP) was purchased from Cayman Chemical (Michigan, UA). Deoxyadenosine, deoxyguanosine, and methylcellulose were purchased from FUJIFILM Wako Pure Chemical Corporation (Osaka, Japan). 2′‐deoxycytidine 5′‐monophosphate (dCMP), deoxycytidine, and thymidine were purchased from Tokyo Chemical Industry (Tokyo, Japan). Dinucleotides and their 5′‐monophosphate derivatives were custom‐synthesized by Eurofins (Tokyo, Japan). All other chemicals and reagents were of the highest purity available and purchased from commercial sources.

### 2.2. Animals

Seven‐week‐old ICR male mice were purchased from Sankyo Labo Service Co. (Tokyo, Japan). Mice were housed under pathogen‐free conditions at a controlled temperature (21°C–25 °C) and subjected to a 12 h light per dark cycle. The lights were turned on from 8:00 to 20:00, and food and water were provided *ad libitum*. The animals were cared for in strict compliance with the guidelines outlined in the National Institutes of Health Guide for the Care and Use of Laboratory Animals. All animal procedures used in this work were approved by the Kanazawa University Animal Care Committee (Permit Number: AP‐183968).

### 2.3. Oral Administration of a Mixture of Nucleic Acids

Seven‐week‐old ICR male mice were fed a chow diet for a week and subsequently fasted for 15 h. The mice were then orally administered either saline (vehicle), omDNA (2 g/kg), HD‐omDNA (2 g/kg), a deoxyribonucleotide (dNT) mixture (500 μmol/kg of dAMP, dGMP, dCMP, and dTMP), adeoxyribonucleoside (dNS) mixture (500 μmol/kg of deoxyadenosine, deoxyguanosine, deoxycytidine, and thymidine), deoxycytidine (500 μmol/kg), and thymidine (500 μmol/kg). The dose of the dNT mixture was adjusted to match the sum of individual dNT content present in the omDNA, where the oral dose of omDNA was estimated by assuming 3 g per day as the amount of food intake in mice and a daily mouse diet containing 2.5% omDNA when improving learning and memory [4]. The dose of the dNS mixture was set to be equivalent to that of the dNT mixture.

### 2.4. Co‐Administration of a Xanthine Oxidase (XO) Inhibitor

Mice were fasted for 15 h and orally administered febuxostat (FEB) (10 mg/kg) as an XO inhibitor or vehicle (0.5% w/v methylcellulose) as a control. One hour later, the mice were orally administered either HD‐omDNA (2 g/kg), deoxyadenosine (500 μmol/kg), deoxyguanosine (500 μmol/kg), or saline (vehicle).

### 2.5. Plasma Sampling

Blood samples were collected from the tail vein at 0, 0.25, 0.5, 1, 3, and 6 h after oral administration. In a different group of mice, blood samples were collected from the portal vein and abdominal aorta under isoflurane anesthesia 5, 15, 30, and 60 min after oral administration. To prevent metabolic conversion by XO during sample processing, 4 μL of 50 μM FEB dissolved in DMSO was added to each blood sample to achieve a final concentration of 10 μM. Blood samples were immediately centrifuged at 3194 ×*g* for 5 min to obtain plasma. The collected plasma samples were stored at −80 °C until analysis.

### 2.6. Synthesis of ^13^C^15^N‐dCTP–Labeled DNA

PCR amplifications were carried out using GoTaq Flexi DNA Polymerase (Promega, WI, USA) on a 50 μL scale which included 10 μL 10× PCR reaction buffer, 1 μL pcDNA3 vector encoding mouse deoxycytidine kinase (mDck) as a DNA template, 1.5 μL (10 μM) DNA primers shown in Supporting Table 1, 0.2 μL Taq polymerase, 3 μL (25 mM) MgCl_2_, and 5 μL (2 mM) of dNTPs. The mixture was first treated with an initial denaturation of 2 min at 95 °C. The amplification was then performed for 40 cycles of 30 s at 95 °C for denaturation, 120 s at 55 °C for annealing, and 60 s at 72 °C for extension, followed by a final extension at 72 °C for 5 min. After PCR amplification, 1/10 volume of 3 M sodium acetate was added, followed by 2.5 volumes of prechilled 100% ethanol. The mixture was then gently inverted and incubated on ice for 1 h. After centrifugation at 20,000 ×*g* for 15 min at 4 °C, the supernatant was carefully removed, and 5 mL of 70% ethanol was added. A second centrifugation was performed under the same conditions, followed by the removal of the supernatant. The DNA pellet was air‐dried at room temperature and dissolved in 480 μL of nuclease‐free water to obtain ^13^C^15^N‐dCTP–labeled DNA.

### 2.7. Transport Experiments in the Ussing‐type Chamber Method

An Ussing‐type chamber, previously described by Shimizu et al. [15], was used, with minor modifications. Briefly, tissue sheets of mice fasted for 18 h, consisting of the mucosa and most of the muscularis mucosa, were mounted vertically in an Ussing‐type chamber that provided an exposed area of 0.25 cm^2^. The volume of bathing solution on each side was 2 mL, and the temperature was maintained at 37 °C. HD‐omDNA was dissolved in transport buffer (pH 6.0) at a final concentration of 10 mg/mL. The dNT mixture, prepared by mixing dAMP, dGMP, dCMP, and dTMP at a final concentration of 2 mM each, and the dNS mixture, prepared by mixing deoxyadenosine, deoxyguanosine, deoxycytidine, and thymidine at 2 mM each, were diluted with pH 6.0 transport buffer. The concentration of the dNT mixture was set to match the sum of individual dNT concentrations in HD‐omDNA, and the dNS mixture was set at equivalent molar concentrations to the dNT mixture. In separate experiments, deoxycytidine, thymidine, deoxyadenosine, and deoxyguanosine were dissolved at a final concentration of 2 mM in pH 6.0 transport buffer. ^13^C^15^N‐labeled DNA and ^13^C^15^N‐dCTP were dissolved in pH 6.0 transport buffer to a final concentration of 0.5 mg/mL and 500 μM, respectively. These samples were applied at the apical side, and at the designated time points, 200 μL of buffer was collected from the basolateral side (pH 7.4) and replaced with an equal volume of fresh pH 7.4 transport buffer. Additionally, 10 μL was also sampled from the apical side at the designated time points. In XO inhibition studies, HD‐omDNA was dissolved in pH 6.0 transport buffer containing 0 or 50 nM FEB, and the buffer on the basolateral side was also supplemented with FEB. All collected samples were stored at −30 °C until analysis.

### 2.8. Measurement of Nucleic Acid Monomers by Liquid Chromatography‐Mass Spectrometry (LC–MS/MS)

Plasma and buffer samples were mixed with 80% acetonitrile (AcCN) or 70% methanol (MeOH), respectively, each containing 100 nM 5‐deoxy‐5‐fluorocytidine as an internal standard. The samples were then centrifuged twice at 21,500 ×*g* for 10 min at 4 °C. The resulting supernatant was subjected to LC–MS/MS analysis. For the samples processed with 70% MeOH, an additional dilution with distilled water was performed to obtain a 20% MeOH solution prior to analysis. The nucleic acid monomer concentration in the plasma was measured using a triple quadrupole mass spectrometer with electrospray ionization coupled with a LCMS (LCMS‐8040; Shimadzu, Kyoto, Japan). Chromatography was performed using stepwise gradient elution (flow rate: 0.4 mL/min) for all measurements. For quantification of cytosine, thymine, uracil, and uric acid, Scherzo SM‐C18 (3 μm particle size, 3.0 × 100 mm column dimensions; Imtakt, Kyoto, Japan) was used, and chromatogram conditions were as follows: 0–0.3 min: 99% A/1% B; 0.3–2.3 min: 99% A/1% B to 70% A/30% B; 2.3–3.0 min: 70% A/30% B to 5% A/95% B; 3.0–4.2 min: 5% A/95% B; 4.2–4.4 min; 5% A/95% B to 99% A/1% B; 4.4–5.5 min 99% A/1% B (A: water containing 0.1% formic acid; B: acetonitrile containing 0.1% formic acid). Column and oven temperatures were 50 °C and 60 °C, respectively. The multiple reaction monitor was set as 111.90 to 95.05 m/z for cytosine, 127.10 to 110.00 m/z for thymine, 113.10 to 69.90 m/z for uracil, 246.00 to 129.95 m/z for 5‐deoxy‐5‐fluorocytidine in the positive‐ion mode. For quantification of other nucleic acid monomers, SeQuant ZIC‐cHILIC (3 μm, 100Å 150 × 2.1 mm, Sigma‐Aldrich, St. Louis, MA, USA) was used, and chromatogram conditions were as follows: 0–1.0 min: 100% B; 1.0–5.0 min: 100% B to 25% A/75% B; 5.0–6.0 min: 25% A/75% B to 30% A/70% B; 6.0–8.0 min: 30% A/70% B; 8.0–8.2 min: 70% A/30% B to 100% B; 8.2–11.0 min: 100% B (A: 80% water/20% acetonitrile containing 5 mM ammonium acetate, 5 mM ammonium formate, and 0.2% formic acid; B: 5% water/95% acetonitrile containing 1 mM ammonium acetate, 1 mM ammonium formate, and 0.2% formic acid). Column and oven temperatures were 50°C and 60 °C, respectively. The multiple reaction monitor was set as 332.15 to 136.05 m/z for dAMP, 347.95 to 134.85 m/z for dGMP, 307.95 to 112.00 m/z for dCMP, 322.90 to 81.00 m/z for dTMP, 308.90 to 81.00 m/z for dUMP, 252.00 to 135.95 m/z for deoxyadenosine, 268.00 to 151.95 m/z for deoxyguanosine, 253.00 to 137.00 m/z for deoxyinosine, 228.00 to 112.05 m/z for deoxycytidine, 243.00 to 127.05m/z for thymidine, 228.95 to 113.00 m/z for deoxyuridine, 268.00 to 135.90 m/z for adenosine, 284.00 to 152.05 m/z for guanosine, 269.20 to 137.00 m/z for inosine, 284.95 to 153.00 m/z for xanthosine, 244.00 to 112.00 m/z for cytidine, 245.00 to 113.10 m/z for uridine, 136.10 to 119.00 m/z for adenine, 151.90 to 134.95 m/z for guanine, 137.10 to 55.10 m/z for hypoxanthine, and 246.00 to 129.95 m/z for 5‐deoxy‐5‐fluorocytidine in the positive‐ion mode and 151.10 to 108.05 m/z for xanthine in the negative‐ion mode.

### 2.9. Measurement of Dinucleotides by LC–TOF/MS

Plasma and buffer samples were mixed with 80% acetonitrile containing 100 nM 5‐deoxy‐5‐fluorocytidine as an internal standard. The samples were then centrifuged twice at 21,500 ×*g* for 10 min at 4 °C. The resulting supernatant was subjected to LC–TOF/MS analysis. Dinucleotide concentrations were measured using an ACQUITY Arc‐System and Xevo G2 Q‐TOF (Waters, Milford, MA, USA). Mass scanning was conducted over the *m/z* range of 100–1000. Chromatography was performed using stepwise gradient elution (flow rate: 0.3 mL/min) for all measurements. Shodex HILICpak VN‐50 2D (5 μm particle size, 150 × 2.0 mm column dimensions, Showa Denko, Tokyo, Japan) was used, and chromatogram conditions were as follows: 0–0.5 min: 1% A/99% B; 0.5–10.5 min: 1% A/99% B to 60% A/40% B; 10.5–12.0 min: 60% A/40% B; 12.0–13.0 min: 60% A/40% B to 1% A/99% B; 13.0–20.0 min; 1% A/99% B (A: water containing 50 mM ammonium formate; B: 5% water/95% acetonitrile containing 10 mM ammonium formate). Column and oven temperatures were 50°C and 60 °C, respectively. Quantification based on the exact mass was performed using TargetLynx software (Waters).

### 2.10. Data Analysis

Data are expressed as mean ± standard deviation (S.D.). The area under the concentration (AUC)–time curve was calculated using the trapezoidal rule with Microsoft Excel (Microsoft Corp., Redmond, WA). The statistical significance of differences was determined using Student’s *t*‐test or repeated measures analysis of variance (ANOVA), followed by Dunnett’s multiple comparison tests for appropriate post hoc analysis. *p* < 0.05 was regarded as denoting a significant difference.

## 3. Results

### 3.1. Plasma Concentration Profiles of Nucleic Acid Monomers Following Oral Administration of HD‐omDNA

**FIGURE 1:**
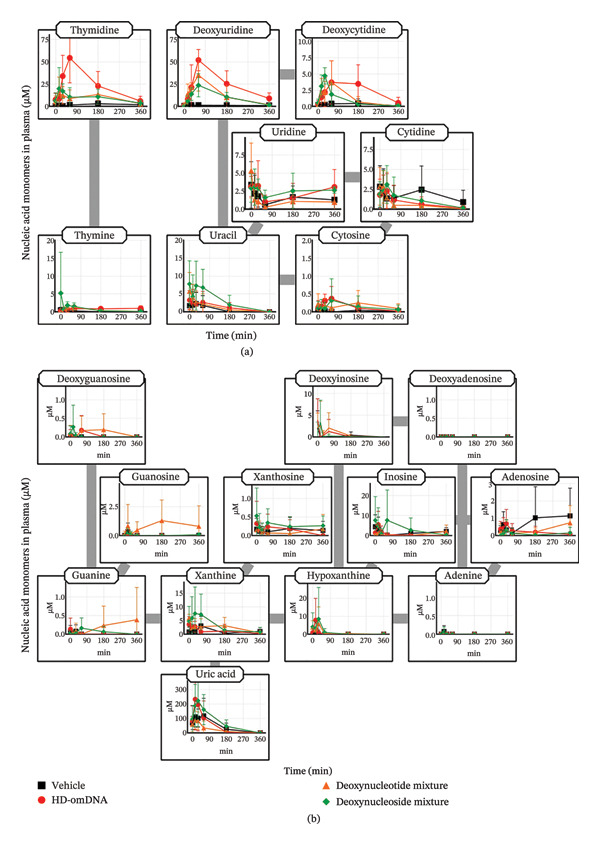
Plasma concentration profiles of nucleic acid monomers after oral administration of HD‐omDNA and a mixture of equivalent amounts of dNTs and dNSs. Vehicle, HD‐omDNA (2 g/kg), a mixture of dNTs (500 µmol/kg of dAMP, dGMP, dCMP, and dTMP), and a mixture of dNSs (500 µmol/kg of deoxyadenosine, deoxyguanosine, deoxycytidine, and thymidine) were orally administered to mice, and tail venous plasma samples were collected at designated times, followed by measurement of the concentrations of (a) pyrimidine and (b) purine nucleic acid monomers using LC–MS/MS. Data are presented as mean ± S.D. (n =5). The AUC values and their statistical difference are provided in Supporting Table 2.

To clarify the molecular forms absorbed into the systemic circulation after oral administration of HD‐omDNA, the plasma concentration profiles of nucleic acid monomers were examined. As a control experiment, these profiles were examined after oral administration of a mixture of dNT, dNS, and vehicle alone. Oral administration of HD‐omDNA resulted in significantly higher AUC values of pyrimidine dNS (thymidine, deoxyuridine, and deoxycytidine) compared to the vehicle administration group (Figure 1(a), Supporting Table 2). In contrast, the plasma concentrations of other pyrimidine NS and bases remained almost unchanged (Figure 1(a)). Notably, the AUC of thymidine and deoxyuridine in the HD‐omDNA group was significantly higher than those in the dNT and dNS groups (Figure 1(a), Supporting Table 2). The time to peak (*T*_max_) of thymidine in the HD‐omDNA group was ∼1 h, which was delayed compared to that in the dNT and dNS groups (Figure 1(a)).

To investigate the absorption properties of longer DNA fragments, omDNA, which consists of 2–20 oligodeoxyribonucleotide bases, was orally administered, and the plasma concentration profiles of nucleic acid monomers were compared with those after oral administration of HD‐omDNA, which consists of one to four oligodeoxyribonucleotides [4]. The AUC of thymidine and deoxyuridine tended to increase following the administration of omDNA, but the AUC value of deoxycytidine in the omDNA group was significantly lower than that in the HD‐omDNA group (Supporting Figure 1, Supporting Table 2), indicating that shorter oligoDNAs exhibited greater efficiency of absorption.

No purine nucleic acid monomers showed a significant increase in plasma concentration compared to that in the vehicle group following oral administration of HD‐omDNA, dNT, and dNS mixtures, except that the AUC value of uric acid tended to be higher after dNS mixture administration compared to that in the vehicle group (Figure 1(b), Supporting Table 2).

### 3.2. Plasma Concentration Profiles of Nucleic Acid Monomers Following Oral Administration of Each Pyrimidine Deoxynucleoside

**FIGURE 2:**
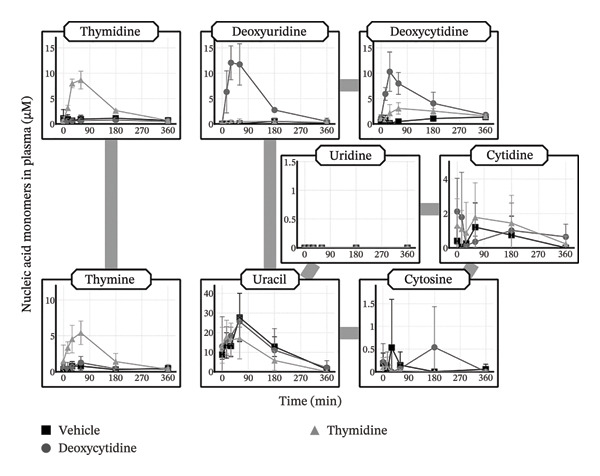
Plasma concentration profiles of pyrimidine nucleic acid monomers after oral administration of pyrimidine deoxynucleoside. Deoxycytidine (500 µmol/kg), thymidine (500 µmol/kg), or vehicle was orally administered to mice, and tail venous plasma samples were collected at designated times, followed by measurement of the concentrations of pyrimidine nucleic acid monomers using LC–MS/MS. Data are presented as mean ± S.D. (n = 4). The AUC values and their statistical difference are provided in Supporting Table 3.

To examine whether the pyrimidine deoxynucleosides that appeared in the plasma following oral administration of HD‐omDNA were at least partially derived from the intestinal absorption of each deoxynucleoside, each pyrimidine deoxynucleoside (thymidine or deoxycytidine) was orally administered individually. Oral administration of thymidine led to an elevation in the AUC of both thymidine and the pyrimidine base thymine, whereas oral administration of deoxycytidine resulted in a significantly higher AUC of both deoxyuridine and deoxycytidine compared to the vehicle group (Figure 2, Supporting Table 3).

### 3.3. Gastrointestinal Absorption of Pyrimidine Deoxyribonucleosides and Bases Assessed by Portal Vein Sampling Following Oral Administration of HD‐omDNA

**FIGURE 3:**
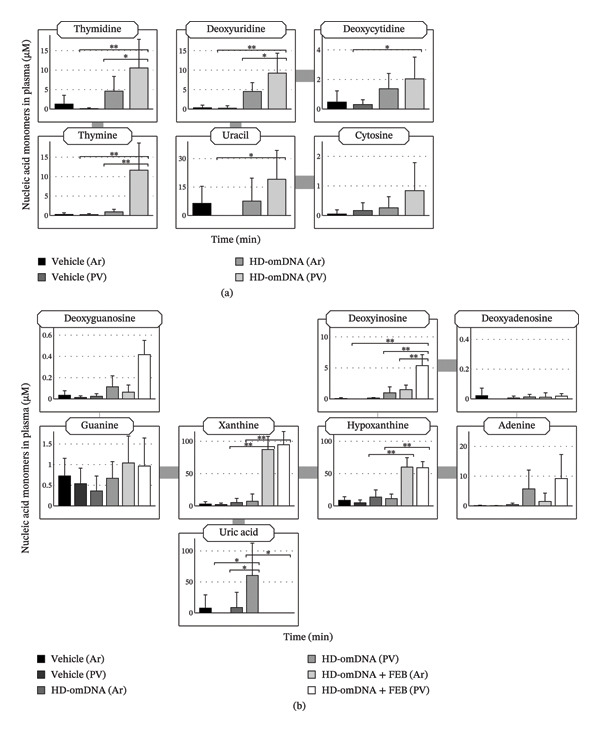
Aorta and portal vein concentrations of (a) pyrimidine and (b) purine nucleic acid monomers after oral administration of HD‐omDNA or vehicle alone. HD‐omDNA (2 g/kg) or vehicle was orally administered to mice, and arterial (Ar) and portal venous (PV) plasma samples were collected 15 min after administration. In panel (b), the XO inhibitor febuxostat (FEB, 10 mg/kg) or vehicle alone was orally administered 1 h before HD‐omDNA administration. The concentrations of (a) pyrimidine and (b) purine nucleic acid monomers were measured using LC–MS/MS. Data are presented as mean ± S.D. (n = 8). ∗*p* < 0.05, ∗∗*p* < 0.01.

To directly investigate the intestinal absorption of pyrimidine deoxynucleosides, both portal vein and arterial plasma concentrations of pyrimidine deoxynucleosides and bases were simultaneously measured after oral administration of HD‐omDNA. The portal vein concentrations of thymidine, deoxyuridine, and thymine were significantly higher than their arterial concentrations in the HD‐omDNA group, whereas the portal vein concentrations of thymidine, deoxyuridine, deoxycytidine, thymine, and uracil in the HD‐omDNA group were higher than those in the vehicle group (Figure 3(a)), supporting the gastrointestinal absorption of at least thymidine, deoxyuridine, and thymine after oral administration of HD‐omDNA. Administration of HD‐omDNA did not significantly change the portal vein plasma concentrations of other nucleosides (cytidine and uridine) or pyrimidine dNTs (dCMP and dTMP) compared with the vehicle group.

### 3.4. Effect of a XO Inhibitor on Plasma Concentration of Purine Nucleic Acid Monomers Following Oral Administration of HD‐omDNA and Each Purine Deoxynucleoside

**FIGURE 4:**
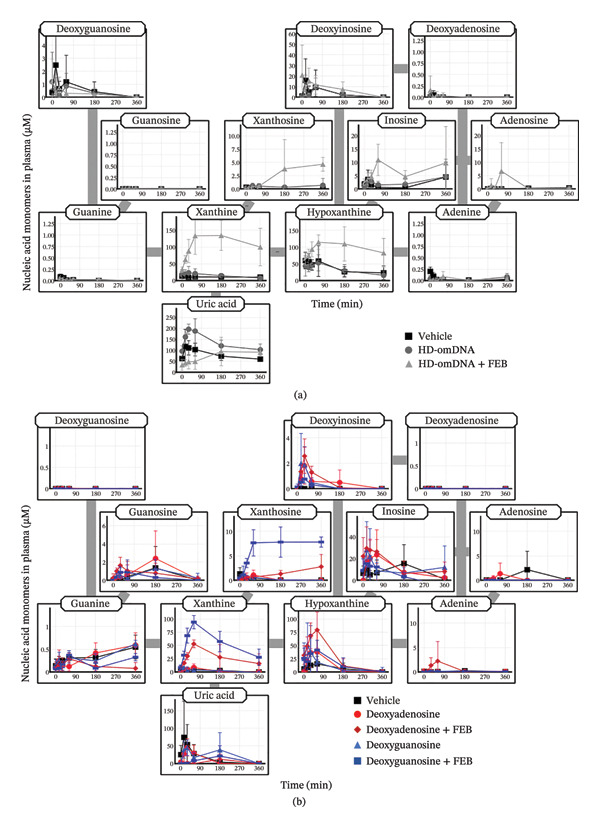
Effect of an XO inhibitor on plasma concentration of purine nucleic acid monomers following oral administration of HD‐omDNA and purine deoxynucleosides. (a) Vehicle or HD‐omDNA (2 g/kg) was orally administered 1 h after oral administration of FEB (10 mg/kg) or vehicle alone in mice. Tail venous plasma samples were collected at designated times, and the concentration of purine nucleic acid monomers was measured using LC–MS/MS. Data are presented as mean ± S.D. (n = 4). (b) Vehicle, deoxyadenosine (500 µmol/kg), or deoxyguanosine was orally administered 1 h after oral administration of FEB (10 mg/kg) or vehicle alone in mice. Tail venous plasma samples were collected at designated times, and the concentration of purine nucleic acid monomers was measured using LC–MS/MS. Data are presented as mean ± S.D. (n = 4). The AUC values and their statistical difference are provided in Supporting Table 4.

No detection of the increase in purine nucleic acid monomers (Figure 1(b)) may result from their rapid degradation, and therefore FEB, a XO inhibitor that blocks the conversion of hypoxanthine to xanthine and xanthine to uric acid in the purine degradation pathway, was administered prior to the administration of HD‐omDNA. Co‐administration of FEB led to an elevation in the AUC values of xanthosine, inosine, hypoxanthine, and xanthine in the HD‐omDNA group (Figure 4(a), Supporting Table 4A). A similar inhibition study was performed after the oral administration of each purine deoxynucleoside, including deoxyadenosine and deoxyguanosine. Co‐administration of FEB resulted in a significantly higher AUC value of xanthine after oral administration of deoxyadenosine (Figure 4(b), Supporting Table 4B), whereas the AUC values of xanthine and xanthosine were significantly elevated by FEB co‐administration in the deoxyguanosine group (Figure 4(b), Supporting Table 4B).

To directly examine the intestinal absorption of each nucleic acid monomer, the portal vein and arterial plasma concentrations were measured after HD‐omDNA administration. In the HD‐omDNA group without FEB, the portal vein concentration of uric acid alone was significantly higher than that in arterial plasma, whereas co‐administration of FEB resulted in a higher portal vein concentration of deoxyinosine compared to its arterial concentration (Figure 3(b)).

### 3.5. Intestinal Membrane Permeability of Nucleic Acid Monomers Assessed in the Ussing‐Type Chamber

**FIGURE 5:**
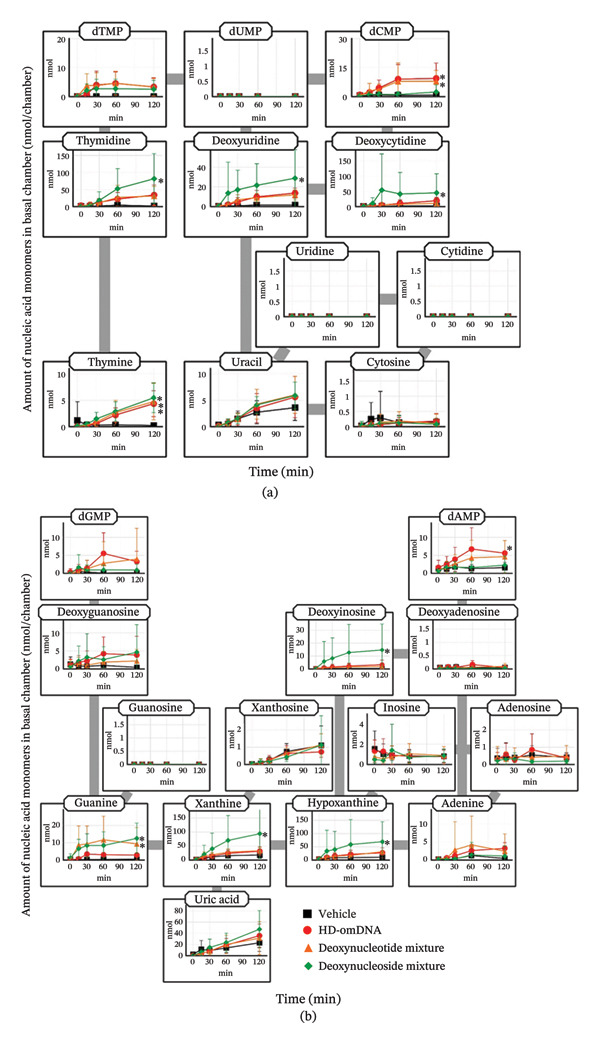
Intestinal permeability of nucleic acid monomers after addition of oligodeoxyribonucleotides, and a mixture of dNTs and dNSs to the apical side of the small intestinal Ussing‐type chamber. Vehicle, HD‐omDNA (10 mg/mL), a mixture of dNTs (2 mM dAMP, dGMP, dCMP, and dTMP), or a mixture of dNSs (2 mM deoxyadenosine, deoxyguanosine, deoxycytidine, and thymidine) was added to the apical side in an Ussing‐type chamber. The amounts of (a) pyrimidine and (b) purine nucleic acid monomers on the basal side were measured using LC–MS/MS. Data are presented as mean ± S.D. (n = 11). ∗Statistical significance was assessed at the final time point, and statistically significant differences compared with the vehicle group are indicated by asterisks (*p* < 0.05).

To directly evaluate the membrane permeability of nucleic acid monomers in the small intestine, an Ussing‐type chamber assay was performed using isolated small‐intestinal tissues. The amount of thymidine and deoxyuridine on the basal side significantly increased in a time‐dependent manner after the addition of the dNS mixture and tended to increase in the HD‐omDNA and dNT groups to the apical side compared with the vehicle group, whereas the appearance of deoxycytidine in the dNS group was also significantly higher than that in the vehicle group (Figure 5(a)). Thymine was significantly increased in all three groups compared with the vehicle group (Figure 5(a)). In the HD‐omDNA and dNT groups, the appearance of dCMP was significantly higher than that in the vehicle group (Figure 5(a)), whereas in the HD‐omDNA group, dAMP appeared on the basal side in a time‐dependent manner and was higher than that in the vehicle group and tended to increase in the dNT group (Figure 5(b)); however, dCMP was much lower than that of nucleoside (Figure 5(a)). The appearance of xanthine, hypoxanthine, and deoxyinosine in the dNS group was higher than in the vehicle group, and guanine was significantly increased in the dNT and dNS groups (Figure 5(b)).

On the apical side, the concentration of dNT, such as dTMP, dCMP, dGMP, and dAMP, was almost comparable between the HD‐omDNA and dNT mixture groups over the 120 min period (Supporting Figure 2), suggesting rapid hydrolysis of HD‐omDNA into dNTs. In the HD‐omDNA and dNT mixture group, no pyrimidine deoxynucleosides were found on the apical side. However, all three pyrimidine deoxynucleosides were found only on the apical side of the dNS mixture group (Supporting Figure 2), whereas thymidine and deoxyuridine were found on the basal side in the HD‐omDNA, dNT, and dNS groups (Figure 5(a)), implying that HD‐omDNA could be absorbed in the form of pyrimidine deoxynucleosides.

### 3.6. Intestinal Membrane Permeability of Nucleic Acid Monomers After Addition of Each Deoxyribonucleoside to the Apical Side

**FIGURE 6:**
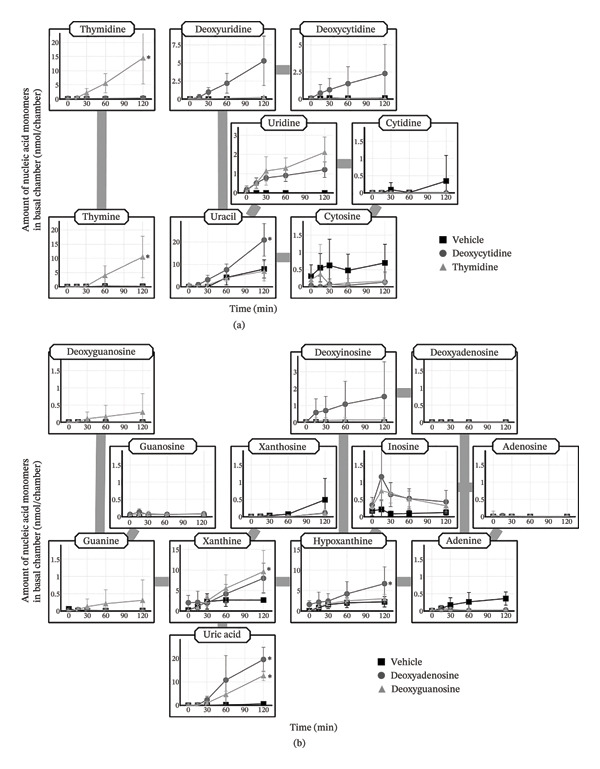
Intestinal permeability of nucleic acid monomers after addition of each deoxyribonucleoside to the apical side of the small intestinal Ussing‐type chamber. (a) Vehicle, deoxycytidine (2 mM), or thymidine (2 mM) was added to the apical side of an Ussing‐type chamber. The amount of pyrimidine nucleic acid monomers in the basal side was measured using LC–MS/MS. Data are presented as mean ± S.D. (n = 4). (b) Vehicle, deoxyadenosine (2 mM), or deoxyguanosine (2 mM) was added to the apical side of an Ussing‐type chamber. The amount of purine nucleic acid monomers in the basal side was measured using LC–MS/MS. Data are presented as mean ± S.D. (n = 4). ∗Statistical significance was assessed at the final time point, and statistically significant differences compared with the vehicle group are indicated by asterisks (*p* < 0.05).

To investigate the intestinal membrane permeability of each deoxynucleoside, the appearance of nucleic acid monomers was examined after the addition of each deoxynucleoside, thymidine, and deoxycytidine, to the apical side. The amount of thymidine and thymine on the basal side significantly increased after the addition of thymidine, whereas the amount of uracil significantly increased, and deoxycytidine and deoxyuridine tended to be higher in the deoxycytidine group (Figure 6(a)). Uridine was not detected in the vehicle group but was detected in the deoxycytidine and thymidine groups (Figure 6(a)). In contrast, in the deoxyguanosine group, the amounts of xanthine and uric acid on the basal side significantly increased (Figure 6(b)). In the deoxyadenosine group, hypoxanthine and uric acid significantly increased, and deoxyinosine and xanthine tended to increase (Figure 6(b)).

### 3.7. Intestinal Membrane Permeability of ^13^C^15^N‐Labeled DNA

**FIGURE 7:**
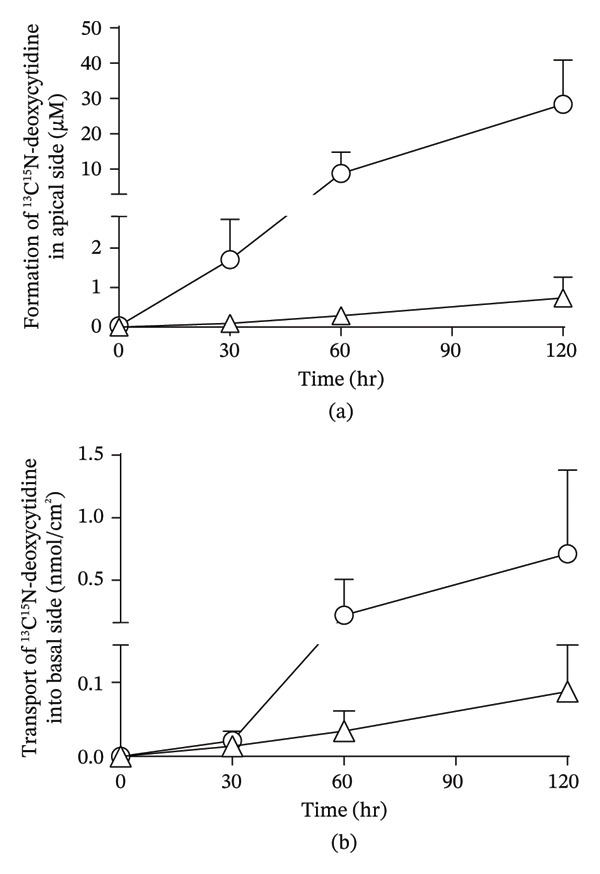
Permeability of ^13^C^15^N‐labeled deoxycytidine across isolated mouse intestinal tissues. ^13^C^15^N‐dCTP (500 µM) (open squares) or ^13^C^15^N‐dCTP labeled mDck (0.5 mg/mL) (open triangles) was added to the apical side of small intestinal tissues in an Ussing‐type chamber. The formation of ^13^C^15^N‐deoxycytidine on the apical side (a) and the appearance of ^13^C^15^N‐deoxycytidine on the basal side (b) were measured using LC–MS/MS. Data are presented as mean ± S.D. (n = 4).

To directly estimate the intestinal membrane permeability of deoxycytidine, ^13^C^15^N‐labeled dCTP and mDck were applied to the apical side, and the appearance of ^13^C^15^N‐labeled deoxycytidine on the apical and basal sides was measured to distinguish it from endogenously synthesized deoxycytidine in the intestinal tissues using the isotope‐labeled compounds. ^13^C^15^N‐labeled deoxycytidine gradually increased on the apical side after the addition of dCTP and mDck (Figure 7(a)), suggesting hydrolysis of these compounds into ^13^C^15^N‐labeled deoxycytidine. In contrast, the appearance of ^13^C^15^N‐labeled deoxycytidine gradually increased on the basal side (Figure 7(b)), suggesting the intestinal membrane permeation of deoxycytidine after the addition of dCTP and mDck. However, because this experiment was performed using only dCTP as one of the representative pyrimidine nucleotides, further studies using other pyrimidine nucleotides are required to determine whether this observation can be generalized.

### 3.8. Detection of Dinucleotides in Portal Vein After Oral Administration of HD‐omDNA

**FIGURE 8:**
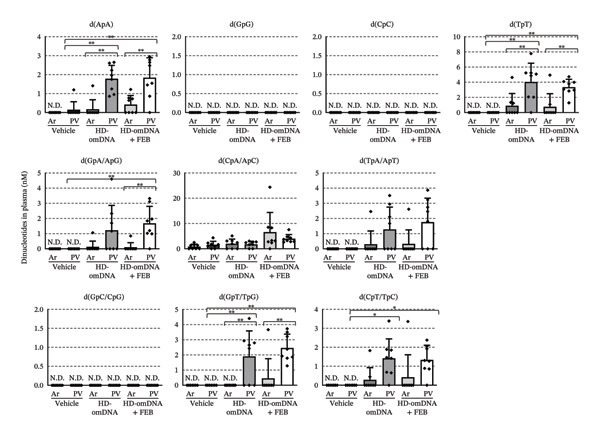
Plasma concentration of dinucleotides in the aorta and portal vein after oral administration of HD‐omDNA. Vehicle or HD‐omDNA (2 g/kg) was orally administered 1 h after oral administration of febuxostat (10 mg/kg) or vehicle alone. Arterial (Ar) and portal venous (PV) plasma samples were collected 15 min after oral administration, and the concentration of dinucleotides was measured using LC–TOF‐MS. Data are presented as mean ± S.D. (n = 8). ∗*p* < 0.05, ∗∗*p* < 0.01. N.D., Under the detection limit (< 10 nM for d(ApA), d(GpG), d(CpC), d(TpT), d(GpA/ApG), d(TpA/ApT), d(GpC/CpG), d(GpT/TpG), and d(CpT/TpC)).

To further investigate the oral absorption of nucleic acid molecules after HD‐omDNA administration, dinucleotides were comprehensively measured in both the portal vein and arterial plasma using LC–TOFMS. Among all 10 combinations of dinucleotides, the concentrations of d(ApA), d(TpT), and d(GpT) in portal vein plasma 15 min after oral administration of HD‐omDNA were significantly higher than those in arterial plasma and portal vein plasma after vehicle administration (Figure 8). However, the absolute values of the concentration found in the portal vein (1.78 nM for d(ApA), 4.03 nM for d(TpT), and 1.90 nM for d(GpT), Figure 8) were substantially lower than those observed for pyrimidine dNSs, bases, or uric acid (Figure 3). In addition, portal vein d(CpT) concentration in the HD‐omDNA group was significantly higher than that in the vehicle group (Figure 8). In contrast, d(GpG), d(CpC), and d(CpG) levels were below the detection limit (Figure 8). Dinucleotides bearing terminal phosphate groups were not detected in either the portal vein or arterial plasma. Although inhibition of XO by FEB was examined, as these dinucleotides include purine nucleotides that may be subject to purine metabolism, no apparent effect of FEB was observed (Figure 8).

### 3.9. Intestinal Membrane Permeability of Dinucleotides After Addition of HD‐omDNA

**FIGURE 9:**
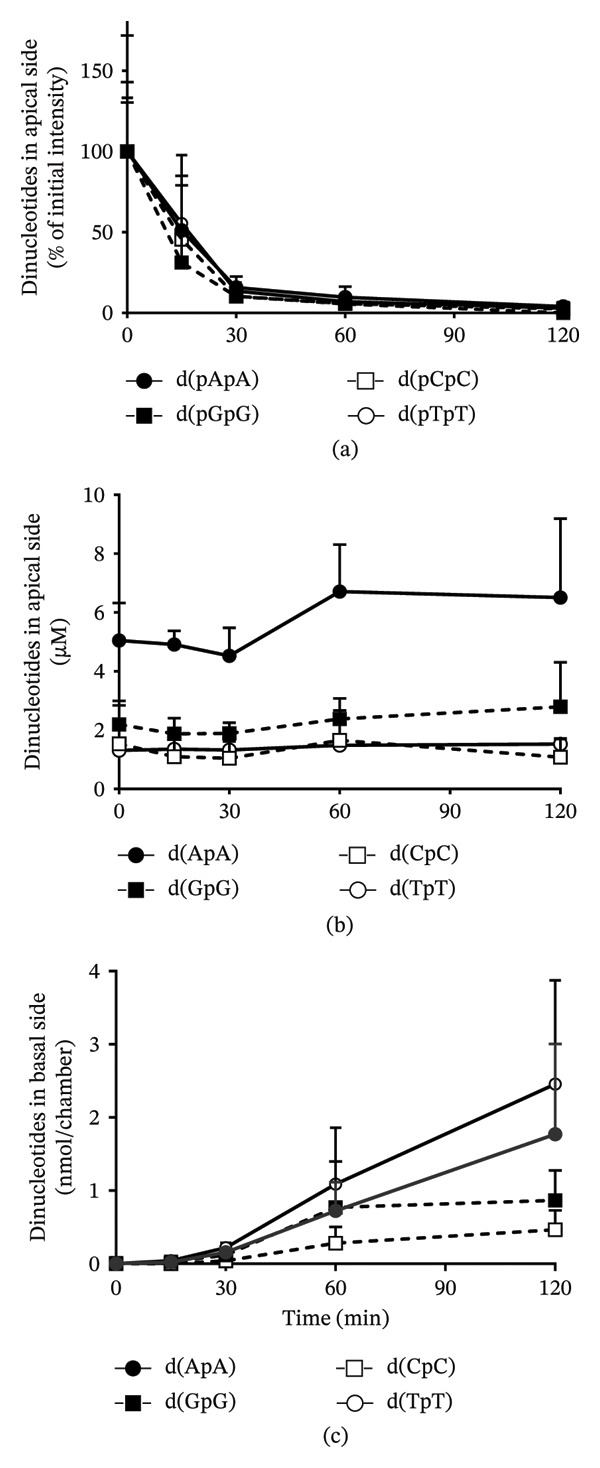
Permeability of dinucleotides across isolated mouse intestinal tissues. HD‐omDNA (10 mg/mL) was added to the apical side of an Ussing‐type chamber. Phosphorylated (a) and dephosphorylated (b, c) dinucleotide on the apical (a, b) and basal (c) sides were measured using LC–TOF‐MS. Data are presented as mean ± S.D. (n = 6). Note that phosphorylated dinucleotides were detected on the apical but not the basal sides.

To evaluate the membrane permeability of dinucleotides in the small intestine, the appearance of dinucleotides on both the apical and basal sides was measured after applying HD‐omDNA to the apical side in an Ussing‐type chamber. On the apical side, phosphorylated dinucleotides such as d(pApA), d(pGpG), d(pCpC), and d(pTpT) were detected and decreased in a time‐dependent manner (Figure 9(a)), whereas their dephosphorylated forms, with particularly high levels of d(ApA) were present throughout the incubation period (Figure 9(b)). On the basal side, dephosphorylated dinucleotides gradually increased, with particularly high levels of d(TpT) and d(ApA) (Figure 9(c)), supporting their potential membrane permeability in the small intestine.

## 4. Discussion

To our knowledge, this is the first comprehensive quantification and characterization of the absorbed forms of nucleic acids following oral administration of oligoDNA preparations, such as HD‐omDNA. Following oral administration of HD‐omDNA, the AUC of all three pyrimidine deoxynucleosides, thymidine, deoxyuridine, and deoxycytidine, increased (Figure 1(a), Supporting Table 2). In addition, the portal vein concentration of these three pyrimidine deoxynucleosides in the HD‐omDNA group was significantly higher than their arterial concentration or portal vein concentration in the vehicle control group (Figure 3(a)), suggesting that these three molecular forms are absorbed in the small intestine. In addition, using ^13^C^15^N‐labeled dCTP and mDck and the Ussing chamber method, labeled deoxycytidine was detected on the basal side (Figure 7(b)), and a time‐dependent increase was observed on the apical side (Figure 7(a)). The enzyme responsible for the conversion of deoxycytidine to deoxyuridine is cytidine deaminase, which exhibits high activity in the liver and is also expressed in the small intestine [16]. Therefore, deoxycytidine may be metabolized to deoxyuridine within the intestinal lumen and/or liver, and deoxyuridine is also the molecular form absorbed into the circulation. These observations are consistent with the hypothesis that nucleoside transporters expressed in the intestinal epithelium are involved in gastrointestinal absorption [1].

Notably, the AUC of thymidine and deoxyuridine in the HD‐omDNA group was higher than those in the dNT and dNS groups when the doses of dNT and dNS were adjusted to match the total amount of dNTs in HD‐omDNA (Figure 1, Supporting Table 2). Intestinal epithelial cells express various hydrolases that degrade high‐molecular‐weight nucleic acids into mononucleotides and nucleosides within the intestinal lumen [2], whereas HD‐omDNA consists of one to four bases of oligodeoxyribonucleotides [4]. Therefore, this result might be explained by the hypothesis that those hydrolases may facilitate the interaction of nucleosides generated by the hydrolases with the nucleoside transporters which are localized on apical membranes and involved in gastrointestinal absorption of nucleosides. This hypothesis should be validated by further analyses. The Tmax values of thymidine in the HD‐omDNA group were delayed compared to those in the dNT and dNS groups (Figure 1), likely reflecting the requirement for oligodeoxyribonucleotides in HD‐omDNA to undergo enzymatic hydrolysis in the intestinal lumen prior to absorption, resulting in slower absorption compared to preformed dNS. Thus, oligoDNA undergoes enzymatic hydrolysis in the intestinal lumen prior to absorption, resulting in slower but extended absorption compared to dNT or dNS, which requires minimal enzymatic degradation and thereby rapidly passes through the gastrointestinal tract before absorption.

After oral administration of HD‐omDNA, purine‐related nucleic acid monomers, such as deoxyadenosine and deoxyguanosine (dNSs); purine bases, including hypoxanthine and xanthine; and dNTs were not detected (Figure 1(b)). In contrast, uric acid was detected in all the groups (Figure 1(b)), indicating that uric acid is intrinsically abundant at least in the systemic circulation. In addition, a comparison of portal vein and arterial plasma concentrations revealed that upon HD‐omDNA administration, only the uric acid level in the portal vein plasma was higher than that in arterial plasma, suggesting that purine nucleic acids are metabolized into uric acid in the gastrointestinal tract prior to absorption, and uric acid is a molecular form orally absorbed into the systemic circulation (Figure 3(b)). However, because the in vivo experiments in the present study were conducted in mice, species‐specific differences in purine metabolism must be considered. Physiological plasma uric acid concentration has been reported to be 178–360 μM in humans and approximately 50 μM in mice [17, 18]. In rodents, uricase is abundantly expressed in the liver, and it metabolizes uric acid to allantoin; however, it is absent in humans [19]. Therefore, uric acid may be further converted into allantoin in mice. Following HD‐omDNA administration to mice, plasma uric acid levels in the tail vein reached Cmax within 15–30 min and returned to vehicle levels 3 h postdosing (Figures 1(b), 4(a)). In contrast, when humans ingested a protein preparation rich in mononucleotides (equivalent to 1.70 g of nucleic acids), plasma uric acid concentrations remained above fasting levels even at 12 h and returned to baseline only after 24 h [20]. Taken together, these findings indicate that the elimination of uric acid in mice is substantially faster than that in humans, reflecting species‐specific differences in purine catabolism. In the Ussing chamber experiments, deoxyinosine, guanine, xanthine, and hypoxanthine were significantly increased in the basal side in the NS mixture group (Figure 5(b)). It was reported that intestinal microflora can metabolize purine nucleosides [21]. Therefore, we cannot exclude potential involvement of microbial metabolism in the observed nucleoside metabolism, and this should be taken into account when interpreting the results.

In the present study, no increase in purine nucleosides/nucleotides was observed in either the HD‐omDNA or vehicle group (Figure 1), and this led us to consider the possibility that XO activity might hinder the observation of purine nucleosides/nucleotides. The increase in plasma xanthine and hypoxanthine concentrations following FEB administration indicates that orally administered purine nucleic acid monomers are metabolized by XO (Figure 4(a)). In addition, the concomitant increase in plasma xanthosine and inosine levels may imply that the inhibition of XO leads to the accumulation of upstream metabolites in the purine degradation pathway. Thus, DNA‐derived deoxyribonucleotides may be sequentially degraded to hypoxanthine and xanthine, and when further metabolism of these metabolites is hindered, their precursors, such as inosine and xanthosine, also accumulate in the plasma. Nevertheless, we did not examine the effect of FEB in the vehicle‐treated group and therefore cannot exclude the possibility that XO activity may affect basal levels of purine nucleosides and/or nucleotides. Collectively, these results highlight the central role of XO in determining the metabolic fate of dietary purine nucleic acids and underscore the potential for metabolic bottlenecks upstream of uric acid formation. Under conditions of concomitant FEB administration, plasma concentrations of purine bases, such as hypoxanthine and xanthine, were elevated in both the portal vein and arterial plasma (Figure 3(b)), but no clear difference was observed between the two. Deoxyinosine in the portal vein was higher than that in arterial plasma under FEB conditions (Figure 3(b)), indicating that deoxyinosine is absorbed across the intestinal epithelium under the inhibition of XO. However, the plasma concentration of deoxyinosine was approximately one‐tenth that of hypoxanthine and xanthine in both the portal vein and arterial blood (Figure 3(b)), implying the existence of additional molecular forms other than deoxyinosine. Considering that XO activity in the proximal small intestine is at least five times higher than in other organs in mice [22], the present findings support the notion that dietary purine nucleic acids are metabolized to uric acid by intestinal XO before entering systemic circulation. However, the intestinal absorption of other purine‐derived compounds may need to be examined, since dinucleotides containing purine bases such as d(ApA) and d(GpT/TpG) were detected in the portal vein (Figure 8), suggesting that some purine nucleic acids might be absorbed in the form of dinucleotides.

Some dinucleotides in portal vein plasma were higher than those in arterial plasma (Figure 8), together with an increase in their appearance in the apical and subsequently basal sides in the Ussing chamber method (Figure 9), suggesting that dinucleotides are also molecular forms absorbed across the gastrointestinal tract after oral administration of HD‐omDNA. Moreover, dephosphorylated dinucleotides increased on the basal side (Figure 9(c)), suggesting that intestinal absorption occurs predominantly in the dephosphorylated form of the dinucleotides. Among these compounds, absorption was the most efficient for d(TpT), followed by d(ApA) (Figures 8 and 9), indicating a sequence‐dependent nature. Amino acids are absorbed efficiently in the form of di‐ and tripeptides via oligopeptide transporter(s) [23], and nucleic acids may also be absorbed in the form of dinucleotides as a nutritionally efficient means of utilization. Indeed, previous studies have reported the detection of polynucleotide fragments in the blood of mice following the ingestion of high‐molecular‐weight DNA [24], as well as the transport of DNA fragments across Caco‐2 cells [25]. Thus, the modes of gastrointestinal absorption of nucleic acids might be diverse and may accompany membrane permeation not only after complete hydrolysis to monomers but also in the form of oligomers or even longer DNA fragments.

Following oral administration of HD‐omDNA, the pyrimidine bases thymine and uracil were significantly elevated in the portal vein compared to those in the vehicle group (Figure 3(a)). Exogenous pyrimidine bases may serve as precursors for nucleotide synthesis via the salvage pathway and are likely incorporated into hepatic nucleic acid metabolism because the liver is a major site of nucleic acid synthesis [2]. Deprivation of dietary nucleotides has been reported to reduce hepatic RNA content and protein synthesis activity [26], indicating that exogenous nucleotide supply may be important for protein synthesis in the liver. In this context, nucleotides synthesized from orally absorbed nucleobases via the salvage pathway may contribute to sustaining hepatic nucleic acid and protein synthesis.

In the present study, the intestinal absorption of oligoDNA in mice was examined at a dose of 2 g/kg body weight, whereas the estimated daily intake of nucleic acids in humans is approximately 0.1–1 g/day [6], indicating that the dose range used in our experiments corresponds to 10–100 times the typical human intake. This represents a limitation of the present study, underscoring the need to investigate the absorption and metabolism of nucleic acids at levels relevant to habitual human consumption. Further elucidation of intestinal absorption mechanisms will advance our understanding of their utilization in vivo and may facilitate their application as functional food ingredients or therapeutic agents.

## Author Contributions

Fuyu Hayashi: data curation, validation, visualization, and writing–original draft. Yusuke Masuo: conceptualization, methodology, project administration, supervision, and writing–original draft. Yuki Nishizawa: data curation, formal analysis, investigation, methodology, validation, and visualization. Ayaka Koike: data curation, formal analysis, investigation, methodology, validation, and visualization. Takahiro Ishimoto: project administration. Keisuke Kiriyama: project administration and resources. Mica Fujita: project administration and resources. Keita Sutoh: project administration, resources, and supervision. Yukio Kato: conceptualization, funding acquisition, project administration, supervision, and writing–original draft.

## Funding

This work was partially supported by a Grant in Aid for Challenging Research (Exploratory) provided to YK (No. 17K19482) from the Ministry of Education, Culture, Sports, Science, and Technology of Japan.

## Disclosure

All authors approved the submission.

## Conflicts of Interest

The authors declare no conflicts of interest.

## Supporting Information

Additional supporting information can be found online in the Supporting Information section.

## Supporting information


**Supporting Information** Supporting Figure 1: Plasma concentration of pyrimidine nucleic acid monomers after oral administration of oligodeoxyribonucleotides. Supporting Figure 2: Concentrations of nucleic acid monomers on the apical side after addition of oligodeoxyribonucleotides, deoxynucleotides, and deoxynucleosides to the apical side of small intestinal tissues of an Ussing‐chamber. Supporting Figure 3: Effect of a xanthine oxidase inhibitor on the permeability of nucleic acid monomers after addition of HD‐omDNA. Supporting Table 1: Primer sequences used for PCR. Supporting Table 2: Area under the curve (AUC) of pyrimidine nucleic acid monomers after oral administration of HD‐omDNA, omDNA, and a mixture of equivalent amounts of dNTs and dNSs. Supporting Table 3: AUC of pyrimidine nucleic acid monomers after oral administration of deoxycytidine and thymidine.

## Data Availability

The data that support the findings of this study are available from the corresponding author upon reasonable request.
