# Supplementary material for: Characterization of Gastrointestinal Absorption of Salmon Milt‐Derived Oligodeoxyribonucleic Acids in Mice
Source: J Nutr Metab. 2026 May 29;2026:2183675. doi: 10.1155/jnme/2183675 (PMC13239040; doi:10.1155/jnme/2183675)
Supplement: Supplementary file 1 — Supporting Information Supporting Figure 1: Plasma concentration of pyrimidine nucleic acid monomers after oral administration of oligodeoxyribonucleotides. Supporting Figure 2: Concentrations of nucleic acid monomers on the apical side after addition of oligodeoxyribonucleotides, deoxynucleotides, and deoxynucleosides to the apical side of small intestinal tissues of an Ussing‐chamber. Supporting Figure 3: Effect of a xanthine oxidase inhibitor on the permeability of nucleic acid monomers after addition of HD‐omDNA. Supporting Table 1: Primer sequences used for PCR. Supporting Table 2: Area under the curve (AUC) of pyrimidine nucleic acid monomers after oral administration of HD‐omDNA, omDNA, and a mixture of equivalent amounts of dNTs and dNSs. Supporting Table 3: AUC of pyrimidine nucleic acid monomers after oral administration of deoxycytidine and thymidine. [file JNME-2026-2183675-s001.docx]

**Supplementary Materials**

**
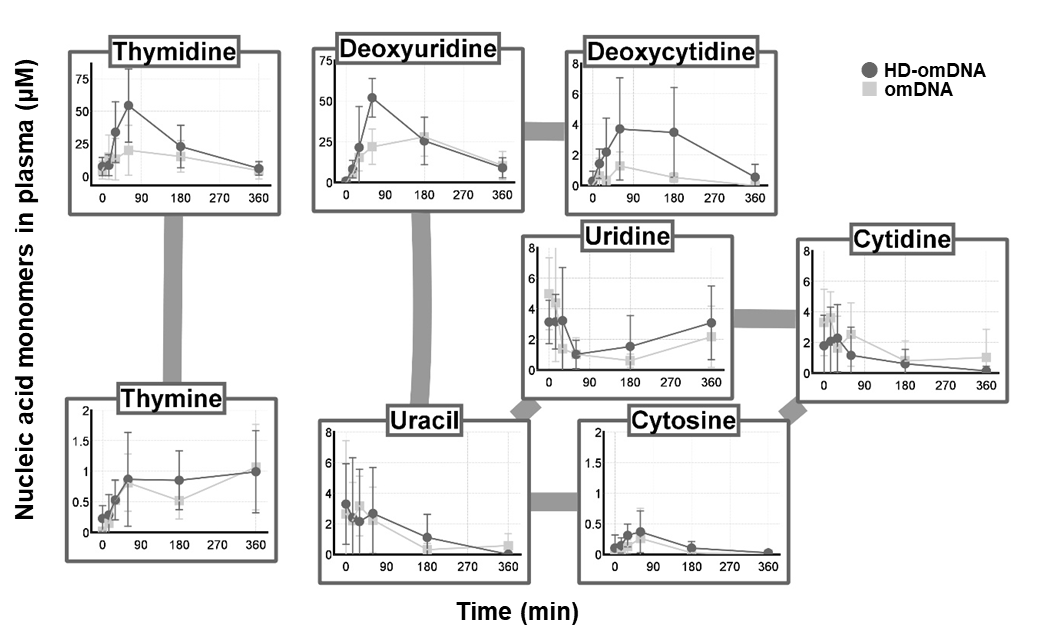
**

**Supplementary Figure 1**

**Plasma concentration of pyrimidine nucleic acid monomers after oral administration of oligodeoxyribonucleotides**

*Oncorhynchus* milt DNA (omDNA; 2 g/kg) was orally administered to mice. Blood samples were collected from the tail vein at designated times, and the plasma concentration of pyrimidine nucleic acid monomers was measured using liquid chromatography–mass spectrometry (LC–MS/MS). Data are presented as mean ± S.D. (n=5). The data after oral administration of hydrolyzed omDNA (HD-omDNA) obtained in Fig. 1 were also shown for comparison. The area under the curve (AUC) values and their statistical difference are provided in Supplementary Table 2.

**
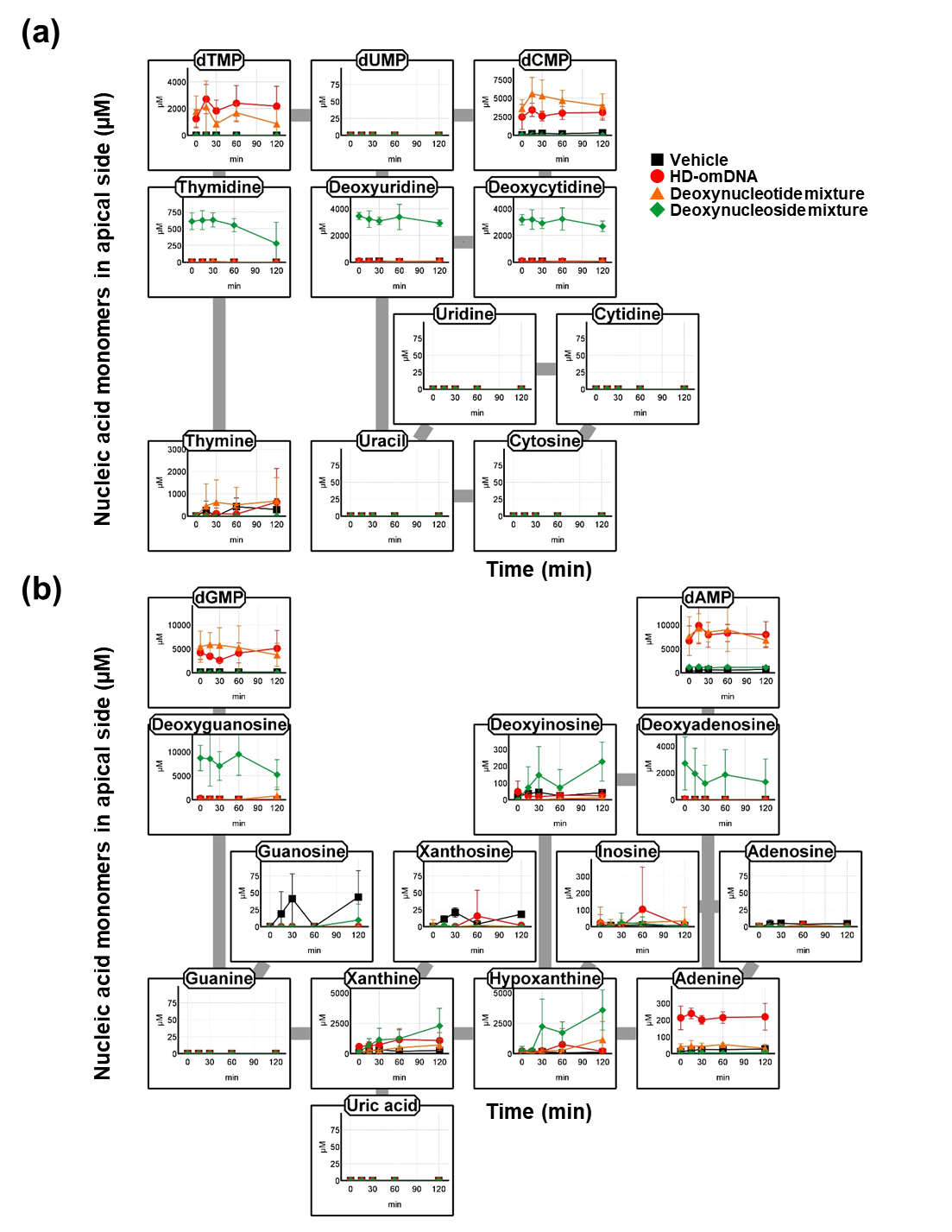
**

**Supplementary Figure 2**

**Concentrations of nucleic acid monomers on the apical side after addition of oligodeoxyribonucleotides, deoxynucleotides, and deoxynucleosides to the apical side of small intestinal tissues of an Ussing-chamber**

Vehicle, HD-omDNA (10 mg/mL), a mixture of deoxynucleotides (dNTs; 2 mM each of dAMP, dGMP, dCMP, and dTMP), or deoxynucleosides (dNSs; 2 mM each of deoxyadenosine, deoxyguanosine, deoxycytidine, and thymidine) were added to the apical side of an Ussing-type chamber. Concentrations of (A) pyrimidine and (B) purine nucleic acid monomers on the apical side were measured using LC–MS/MS. Data are presented as mean ± S.D. (n=6).

**Supplemental Table 1. Primer sequences used for PCR.**

| Primer | Sequence (5′–3′) |
| --- | --- |
| T7 (Forward) | TAATACGACTCACTATAGGG |
| BGH (reverse) | TAGAAGGCACAGTCGAGG |

**Supplementary Table 2. Area under the curve (AUC) of pyrimidine nucleic acid monomers after oral administration of HD-omDNA, omDNA, and a mixture of equivalent amounts of dNTs and dNSs**

|  | AUC(0-6) (ng·h/mL)^a)^ | | | | |
| --- | --- | --- | --- | --- | --- |
|  | Vehicle | HD-omDNA | dNT | dNS | omDNA |
| Thymidine | 3.06±2.46 ×10^3^ | 36.4±17.7 ×10^3^ ^*^ | 14.1±8.4 ×10^3 †^ | 14.0±9.3 ×10^3 †^ | 19.2±13.4 ×10^3^ |
| Deoxyuridine | 1.60±1.49 ×10^3^ | 34.7±9.3 ×10^3^ ^*^ | 19.2±8.8 ×10^3 *†^ | 14.6±4.6 ×10^3 *†^ | 27.3±10.2 ×10^3^ |
| Deoxycytidine | 0.441±0.527 ×10^3^ | 3.49±2.27×10^3 *^ | 1.77±1.13 ×10^3 *^ | 1.36±0.60 ×10^3 *^ | 0.717±0.414 ×10^3 †^ |
| Uridine | 2.07±1.58 ×10^3^ | 2.95±1.18 ×10^3^ | 1.43±1.23 ×10^3^ | 3.60±1.25 ×10^3^ | 2.01±0.98 ×10^3^ |
| Cytidine | 2.57±2.44 ×10^3^ | 1.15±1.11 ×10^3^ | 0.823±0.395 ×10^3^ | 1.71±1.47 ×10^3^ | 2.09±1.22 ×10^3^ |
| Thymine | 0.868±1.155 ×10^2^ | 6.31±1.73 ×10^2^ | 3.91±1.06 ×10^2^ | 5.31±3.21 ×10^2^ | 5.23±1.64 ×10^2^ |
| Uracil | 4.23±5.73 ×10^2^ | 8.85±6.32 ×10^2^ | 7.68±7.61 ×10^2^ | 20.5±16.1 ×10^2^ | 7.20±1.38 ×10^2^ |
| Cytosine | 1.66±2.18 ×10^1^ | 10.1±5.7 ×10^1^ | 11.4±13.9 ×10^1^ | 10.0±12.4 ×10^1^ | 4.88±7.48 ×10^1^ |
| Uric acid | 4.75±4.04 ×10^4^ | 4.67±4.26 ×10^4^ | 2.09±1.64 ×10^4^ | 7.67±4.93 ×10^4^ | 4.68±2.69 ×10^4^ |

a) AUC values of each compound from time 0 to 6 hours were calculated using the trapezoidal method based on the data shown in Figures 1 and S1, and shown as Mean ± S.D. (n=5).

*P < 0.05 vs AUC of vehicle, ^†^P < 0.05 vs AUC of HD-omDNA.

**Supplementary Table 3. AUC of pyrimidine nucleic acid monomers after oral administration deoxycytidine and thymidine.**

|  | AUC(0-6) (ng·h/mL×10^2^)^a)^ | | |
| --- | --- | --- | --- |
|  | Vehicle | deoxycytidine | Thymidine |
| Thymidine | 13.4±3.9 | 9.35±1.31 | 54.0±6.6 ^*^ |
| Deoxyuridine | 3.57±2.54 | 64.8±11.3 ^*^ | 5.78±4.97 |
| Deoxycytidine | 12.6±2.5 | 64.4±16.0 ^*^ | 31.0±1.9 |
| Uridine | N.D. | N.D. | N.D. |
| Cytidine | 8.51±4.12 | 11.4±5.3 | 16.8±9.0 |
| Thymine | 3.46±1.65 | 4.47±3.04 | 17.1±5.1 ^*^ |
| Uracil | 87.4±12.0 | 84.3±31.9 | 53.8±14.5 |
| Cytosine | 0.548±0.551 | 1.67±2.06 | 0.0754±0.0510 |

a) AUC values of each compound from time 0 to 6 hours were calculated using the trapezoidal method based on the data shown in Figure 2 and shown as Mean ± S.D. (n=4).

*P < 0.05 vs AUC of vehicle. N.D., Under detection limit (< 7.33 ng/mL uridine)

**Supplementary Table 4. AUC of purine nucleic acid monomers after oral administration HD-omDNA, deoxyadenosine, and deoxyguanosine** **with or without febuxostat.**

**(A)**

|  | AUC(0-6) (ng·h/mL×10^2^)^a)^ | | |
| --- | --- | --- | --- |
|  | Vehicle | HDomDNA | HD-omDNA+FEB |
| Xanthosine | 0.598±0.250 ×10^2^ | 7.72±4.99 ×10^2^ | 49.0±38.4 ×10^2^ ^*^ |
| Inosine | 2.69±1.82 ×10^3^ | 4.02±3.24 ×10^3^ | 11.5±3.6 ×10^3 *^ |
| Xanthine | 9.76±1.78 ×10^3^ | 13.7±5.0 ×10^3^ | 107±28 ×10^3 *^ |
| Hypoxanthine | 2.87±1.20 ×10^4^ | 2.65±0.32 ×10^4^ | 8.21±2.47 ×10^4 *^ |

**(B)**

|  | AUC(0-6) (ng·h/mL×10^2^)^a)^ | | | | |
| --- | --- | --- | --- | --- | --- |
|  | Vehicle | deoxyadenosine | deoxyadenosine +FEB | deoxyguanosine | deoxyguanosine +FEB |
| Xanthosine | 0.299±0.308 ×10^3^ | 0.461±0.404 ×10^3^ | 2.42±1.22 ×10^3^ | 0.378±0.272 ×10^3^ | 12.1±3.0 ×10^3 *^ |
| Xanthine | 2.18±1.68 ×10^3^ | 2.21±0.76 ×10^3^ | 26.7±11.7 ×10^3 *^ | 1.87±1.22 ×10^3^ | 51.3±10.8 ×10^3 *^ |
| Hypoxanthine | 5.66±5.43 ×10^3^ | 9.51±6.71 ×10^3^ | 23.2±9.5 ×10^3^ | 8.71±6.63 ×10^3^ | 13.3±7.6 ×10^3^ |

a) AUC values of each compound from time 0 to 6 hours were calculated using the trapezoidal method based on the data shown in Figure 4 and shown as Mean ± S.D. (n=4).

*P < 0.05 vs AUC of vehicle.
